# Tissue-Specific Warburg Effect in Breast Cancer and Cancer-Associated Adipose Tissue—Relationship between AMPK and Glycolysis

**DOI:** 10.3390/cancers13112731

**Published:** 2021-05-31

**Authors:** Andjelika Kalezic, Mirjana Udicki, Biljana Srdic Galic, Marija Aleksic, Aleksandra Korac, Aleksandra Jankovic, Bato Korac

**Affiliations:** 1 Department of Physiology, Institute for Biological Research “Sinisa Stankovic”—National Institute of Republic of Serbia, University of Belgrade, 11000 Belgrade, Serbia; andjelika.kalezic@ibiss.bg.ac.rs (A.K.); aleksandra.jankovic@ibiss.bg.ac.rs (A.J.); 2 Department of Anatomy, Faculty of Medicine, University of Novi Sad, 21000 Novi Sad, Serbia; mirjana.udicki@mf.uns.ac.rs (M.U.); biljana.srdic-galic@mf.uns.ac.rs (B.S.G.); 3 Center for Electron Microscopy, Faculty of Biology, University of Belgrade, 11000 Belgrade, Serbia; marija.aleksic@bio.bg.ac.rs (M.A.); aleksandra.korac@bio.bg.ac.rs (A.K.)

**Keywords:** breast cancer, cancer-associated adipose tissue, AMPK, glycolysis, hexokinase, glycogen, obesity

## Abstract

**Simple Summary:**

Specific metabolic phenotypes of breast cancer result from local interactions such as cancer-adipocyte cross-talk and systemic metabolic influences such as obesity. Here we examined key regulatory enzymes involved in glucose metabolism in breast cancer tissue and cancer-associated adipose tissue of normal-weight and overweight/obese premenopausal women in comparison to benign breast tumor tissue and adipose tissue of weight-matched women. We show a simultaneous increase in 5′-AMP-activated protein kinase (AMPK) protein expression with glucose utilization favoring glycolysis and pentose phosphate pathway in breast cancer tissue. In parallel, we show an increased AMPK protein expression with glucose utilization favoring the pentose phosphate pathway in cancer-associated adipose tissue. Moreover, specific features of cancer tissue glycolysis and glycogen metabolism differ between normal-weight and overweight/obese women. The results suggest context-dependent induction of tissue-specific Warburg effect in breast cancer and cancer-associated adipose tissue.

**Abstract:**

Typical features of the breast malignant phenotype rely on metabolic reprogramming of cancer cells and their interaction with surrounding adipocytes. Obesity is strongly associated with breast cancer mortality, yet the effects of obesity on metabolic reprogramming of cancer and cancer-associated adipose tissue remain largely unknown. Paired biopsies of breast tumor tissue and adipose tissue from premenopausal women were divided according to pathohistological analyses and body mass index on normal-weight and overweight/obese with benign or malignant tumors. We investigated the protein expression of key regulatory enzymes of glycolysis, pentose phosphate pathway (PPP), and glycogen synthesis. Breast cancer tissue showed a simultaneous increase in 5′-AMP-activated protein kinase (AMPK) protein expression with typical features of the Warburg effect, including hexokinase 2 (HK 2) overexpression and its association with mitochondrial voltage-dependent anion-selective channel protein 1, associated with an overexpression of rate-limiting enzymes of glycolysis (phosphofructokinase 1—PFK-1) and pentose phosphate pathway (glucose-6-phosphate dehydrogenase—G6PDH). In parallel, cancer-associated adipose tissue showed increased AMPK protein expression with overexpression of HK 2 and G6PDH in line with increased PPP activity. Moreover, important obesity-associated differences in glucose metabolism were observed in breast cancer tissue showing prominent glycogen deposition and higher glycogen synthase kinase-3 protein expression in normal-weight women and higher PFK-1 and glyceraldehyde 3-phosphate dehydrogenase (GAPDH) protein expression in overweight/obese women. In conclusion, metabolic reprogramming of glycolysis contributes to tissue-specific Warburg effect in breast cancer and cancer-associated adipose tissue.

## 1. Introduction

Breast cancer incidence has been rising in recent decades [1], making it the most common malignancy among young women below 40 years of age [2]. Despite continuous advances in diagnostic and therapeutic approaches, premenopausal breast cancer often presents at more advanced stages with worse prognoses than postmenopausal breast cancer [3,4]. Another global health concern, obesity, is taking on epidemic proportions, and accumulating evidence has linked it to breast cancer incidence [5]. Obesity is known to increase the risk of postmenopausal breast cancer, while it seems to decrease the risk of premenopausal breast cancer [6]. Nevertheless, most clinical studies show more aggressive breast cancer phenotypes, higher rates of metastasis, therapeutic resistance, and mortality in both pre- and postmenopausal obese women compared to normal-weight women [7,8,9]. These findings demonstrate that our current understanding of the complex relationship between breast cancer and obesity warrants urgent attention.

Recent studies show that glycolytic reprogramming serves multiple purposes in cancer, including energy production, supply of metabolic intermediates for biosynthetic pathways, posttranslational regulation through glycoprotein synthesis, and redox homeostasis maintenance [10]. Altered glucose metabolism is one of the first recognized features of malignant tumors since Otto Warburg discovered that cancer cells utilize glycolysis leading to lactate production even in the presence of oxygen [11]. Accordingly, this phenomenon was termed the Warburg effect. Later, Pedersen [12] defined hexokinase 2 (HK 2) overexpression and its mitochondrial association with voltage-dependent anion channel (VDAC) as primary signatures of the Warburg effect. Phosphorylation of glucose by hexokinases is the first committed step in glucose catabolism directing it to glycolysis and pentose phosphate pathway (PPP). High glucose uptake and intensified glucose metabolism are partly enabled by HK 2 gene duplication and overexpression [13]. Additionally, association with mitochondrial VDAC releases HK 2 from inhibition by glucose 6-phosphate and provides direct access to ATP, increasing HK 2 activity and promoting prolonged glycolysis [14,15]. Furthermore, the rate-limiting glycolytic enzyme, phosphofructokinase 1 (PFK-1), catalyzes the key regulatory step in glycolysis, acting as a metabolic valve and determining the glycolytic flux [16]. In a parallel metabolic pathway, the rate-limiting enzyme, glucose-6-phosphate dehydrogenase (G6PDH), directs glucose into PPP [17].

Metabolic reprogramming is no longer viewed as an exclusively intrinsic trait of cancer cells. Rather, metabolic reprogramming is a context-dependent and dynamic process resulting from bidirectional interactions between cancer cells and their local and systemic environment [18]. The tumor microenvironment entails specific physical properties, extracellular matrix, secreted factors, and various types of stromal cells [19]. Cancer-associated adipocytes have emerged as valuable contributors to breast cancer development and progression [20]. They were first recognized to stimulate breast cancer cell proliferation through adipokines, proinflammatory cytokines, and growth factors [21]. Currently, cancer-adipose tissue cross-talk is studied as an important example of cancer-stroma metabolic cooperation [22,23]. Adipocytes can engage in intracellular trafficking of many metabolites, including fatty acids, glycerol, ketone bodies, lactate, as well as whole exosomes with cancer cells [24,25]. However, little is known about the mechanisms of adipose tissue metabolic reprogramming that enables such metabolic coupling. Moreover, data on in vivo metabolic signatures of mammary adipose tissue, relevant for breast cancer, is very scarce.

Our previous studies indicated strong lactate-centric cooperation between breast cancer and cancer-associated adipose tissue in premenopausal women. Our data showed that adipose tissue-expressing lactate dehydrogenase B was found in close proximity to cancer tissue rich in lactate and expressing lactate dehydrogenase A [25]. Moreover, we showed that such complementary metabolic reprogramming was underpinned by redox reprogramming favoring increased antioxidant defense levels in both breast cancer and cancer-associated adipose tissue [26].

Our current study aimed to uncover the relationship between key features of glucose metabolism in breast cancer and cancer-associated adipose tissues. We investigated the context-dependent nature of glucose metabolism by undertaking a cross-examination of breast cancer and adipose tissue biopsies from normal-weight and overweight/obese premenopausal women. We analyzed protein expression of key rate-limiting and regulatory enzymes involved in major pathways of glucose metabolism, including glycolysis, PPP, and glycogen metabolism, as well as hallmarks of the Warburg effect such as HK 2 protein expression and cellular localization. Our results reveal parallel increases in 5′-AMP-activated protein kinase (AMPK) protein expression levels in breast cancer and cancer-associated adipose tissue, as well as the induction of tissue-specific Warburg effects. In addition, the results strongly support the concept that obesity can directly influence key features of glucose metabolism in breast cancer tissue of premenopausal women and add to our understanding of metabolic reprogramming in the breast cancer microenvironment.

## 2. Materials and Methods

### 2.1. Patient Recruitment and Tumor Sample Collection

Thirty-six premenopausal women with regular menstrual cycles for at least six months were included in the study. According to the World Health Organization criteria [27], participants were grouped into normal-weight (BMI < 25 kg/m^2^) or overweight/obese (BMI ≥ 25 kg/m^2^). All women were scheduled for surgical removal of benign or malignant breast tumors. Under general balanced anesthesia, two tissue samples were taken from each patient; one of the breast tumor tissue and one of the breast adipose tissue. Breast (mammary) adipose tissue that was proximal to the tumor but did not include the invasive front of the tumor was designated as tumor-associated adipose tissue. Following the histopathological analysis, samples were classified into either benign breast fibroadenomas or malignant breast invasive ductal carcinomas (luminal type A (ER^+^/PR^+^/HER2^−^), thus forming four groups of samples (*n* = 9): benign tumors from normal-weight individuals, benign tumors from overweight/obese individuals, malignant tumors from normal-weight individuals, and malignant tumors from overweight/obese individuals. Each sample of tumor and adipose tissue was cut into two parts for protein expression/localization analyses, either quantitative (western blotting) or cell type/localization (immunohistochemistry/immunofluorescence). For western blotting, one piece of the tissue sample was immediately frozen in liquid nitrogen. Samples were stored at −80 °C until subsequent protein isolation (TRI Regent, Ambion, Austin, TX, USA). For immunohistochemical and immunofluorescent analyses, the second piece of the tissue sample was immediately fixed in 4% paraformaldehyde (PFA) solution and stored in ethanol at 4 °C before downstream histological processing.

### 2.2. Western Blotting

Western blot analysis was done for the determination of protein content according to the previously published protocol [28]. Primary antibodies against 5′-AMP-activated protein kinase, hexokinase 1, hexokinase 2, phosphofructokinase 1, glyceraldehyde-3-phosphate dehydrogenase, glucose-6-phosphate 1-dehydrogenase, glycogen synthase kinase-3, and house-keeping protein β-actin were purchased from Millipore Sigma (Burlington, MA, USA), Santa Cruz (Dallas, TX, USA), and Abcam (Cambridge, UK) and used at the following concentrations; AMPK (1 µg/mL; #07-350SP), HK 1 (1 µg/mL; sc46695), HK 2 (1 µg/mL; ab104836), PFK-1 (1 µg/mL; ab119796), GAPDH (0.2 µg/mL; ab8245), G6PDH (1 µg/mL; ab76598), GSK-3 α/β (1 µg/mL; ab90366), and β-actin (0.5 µg/mL; ab8226). Densitometric analyses were performed on immunoreactive bands using the Image J software (Version v1.53c, National Institutes of Health, Bethesda, MD, USA). The sum of pixel intensities for each band was considered as the total band density for each protein of interest. Subsequently, band density was normalized as the ratio of pixel intensity for the target protein averaged against the loading control (β-actin). For all proteins of interest, protein content was shown as protein expression in arbitrary units (AU) from three independent experiments. Images of whole uncut blots with values of densitometric analysis for each band are shown Figure S1.

### 2.3. Periodic Acid-Schiff Staining

Periodic Acid-Schiff (PAS) staining for the histological detection of glycogen was performed according to the manufacturer’s instructions (Cat. No. 04-163802, Bio Optica, Milano, Italy). To detect glycogen-specific staining, pretreatment with a diastase kit containing α amylase was used according to the manufacturer’s instructions (Cat. No. 04-140805, Bio Optica, Milano, Italy). For each sample, pairs of serial sections were used for PAS staining, where one section was pretreated with diastase and served as a negative control for its respective serial section. Five µm thick paraffin-embedded serial sections were used for the staining.

### 2.4. Immunohistochemistry

Immunohistochemistry was used to confirm cell-specific protein expression and localization of HK 2 in cancer cells and adipocytes in breast tumor and adipose tissue. 4% PFA-fixed samples were embedded into the paraffin and cut into 5µm tick serial sections. The immunolabeling procedure was performed following a previously published protocol [29] using primary antibodies against HK 2 (2 µg/mL; ab104836) and the appropriate secondary antibodies (all purchased from Abcam, Cambridge, UK). Namely, before incubation with antibodies, antigen retrieval was performed in the microwave for 5 min until citrate buffer was brought to the boiling point. Sections were incubated with primary antibodies overnight at 4 °C, rinsed, and incubated with secondary antibodies for 2 h at room temperature the next day. A Dako LSAB Universal Kit was used for immunodetection and visualization (Dako Scientific, Glostrup, Denmark). Finally, hematoxylin was used for counterstaining; sections were mounted with DPX (Sigma-Aldrich, St Louis, MO, USA) and examined with a Leica DMLB microscope (Leica Microsystems, Wetzlar, Austria).

### 2.5. Immunofluorescence and Microscopy

Immunofluorescence and confocal microscopy were used to analyze the cellular localization and colocalization patterns of relevant proteins following a previously published procedure. Briefly, a sequential immunofluorescence assay was performed in the dark on PFA-fixed, paraffin-embedded 5µm tick serial sections [30] using primary antibodies against VDAC (5 µg mL^−1^; ab46545, Abcam), HK 1 (5 µg mL^−1^; ab46545, Abcam), and HK 2 (5 µg mL^−1^; ab46545, Abcam) and the appropriate fluorochrome-conjugated secondary antibodies Alexa Fluor 488 and Alexa Fluor 633 (1:400, A-11029, Alexa Fluor^®^ 488 goat anti-rabbit, and 1:400, A-21052; Alexa Fluor^®^ 633, Life Technologies, Carlsbad, CA, USA). Sytox Orange (1 µL mL^−1^, Thermo Fisher Scientific, Waltham, MA, USA) was used as a counterstain to visualize the nuclei. Mowiol (Polysciences, Eppelheim, Germany) was used as a mounting agent. Leica TCS SP5 confocal laser scanning microscope (Leica Microsystems, Wetzlar, Germany) was used in sequential mode to acquire confocal images and avoid bleed-through between different channels. The same procedure with the omission of primary antibodies was used as a negative control to test the specificity of antibodies.

### 2.6. Statistical Data Analyses

All data were statistically analyzed using GraphPad Prism software (Version 6.01 GraphPad Software, San Diego, CA, USA). All data sets were tested for the normality of distribution using D’Agostino or Pearson’s omnibus normality test. To compare within-group variation, a one-way two-tailed analysis of variance (ANOVA) was applied. After assessing the overall differences by the F test, the comparisons of inter-group differences were evaluated by multiple comparison Tukey’s post hoc test. Statistical significances were considered at *p* < 0.05.

## 3. Results

### 3.1. Breast Cancer Tissue Shows Hallmark Signatures in Major Glucose Metabolic Pathways with Obesity-Related Differences between Normal-Weight and Obese Premenopausal Women

**Figure 1:**
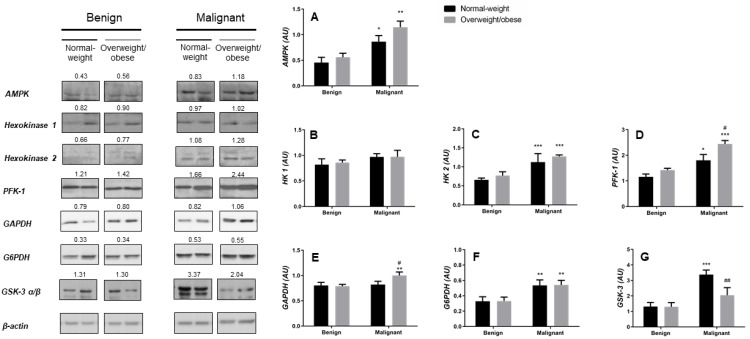
Breast cancer changes in glucose metabolic pathways and their relationship to obesity. Protein expression of 5′-AMP-activated protein kinase (AMPK, **A**), hexokinase 1 (HK 1, **B**), hexokinase 2 (HK 2, **C**), phosphofructokinase (PFK-1, **D**), glyceraldehyde-3-phosphate dehydrogenase (GAPDH, **E**), glucose-6-phosphate 1-dehydrogenase (G6PDH, **F**) and glycogen synthase kinase-3 (GSK-3 α/β, **G**) in benign and malignant breast tumor tissue of normal-weight (black) and overweight/obese (gray) women. The protein content is normalized to β-actin levels and expressed in arbitrary units (AU); mean values of densitometric analysis for each group are shown above respective bands. Two representative band images cut from the same blot are shown for each group (*n* = 9); each band is representative of three pooled samples. Images of whole uncut blots with values of densitometric analysis for each band are shown Figure S1. Bars represent average values ± S.E.M. * Comparison to respective benign counterpart, * *p* < 0.05, ** *p* < 0.01, *** *p* < 0.001. # Comparison to normal-weight malignant counterpart, # *p* < 0.05, ## *p* < 0.01.

Assessment of the protein expression levels of the main energy sensor regulating cellular energy metabolism, AMPK as well as the rate-limiting enzymes within three major pathways of glucose metabolism (glycolysis, pentose phosphate pathway, and glycogen metabolism) revealed profound changes in the energy metabolism of malignant tumor tissue in comparison to benign tumor tissue. Consistent with the high energy demands of cancer tissue, AMPK protein expression was significantly higher in malignant tumor tissue than in benign tumor tissue, irrespective of obesity (Figure 1A). Similarly, the key rate-limiting enzyme of glycolysis and one of the hallmarks of neoplastic transformation, HK 2, was significantly overexpressed in malignant tumor tissues, while HK 1 was constitutively expressed and did not differ between benign and malignant tumor tissues, irrespective of obesity (Figure 1B,C). The main enzymes for the preparatory and terminal phases of glycolysis, PFK-1 and glyceraldehyde-3-phosphate dehydrogenase (GAPDH) showed a similar pattern towards increased expression in malignant tumor tissues (Figure 1D,E). Interestingly, PFK-1 showed an even higher protein expression level in malignant tumor tissues of overweight/obese women compared to malignant tumor tissue of normal-weight women, while GAPDH only showed an increased expression in malignant tumor tissues of overweight/obese women. This observed difference could reflect context-dependent changes in glycolytic fluxes showing a stronger tendency towards pyruvate production in malignant tumor tissue of overweight/obese than in normal-weight premenopausal women. As expected, the protein expression level of the rate-limiting enzyme of pentose phosphate pathway G6PDH (Figure 1F) was significantly higher in malignant tumor tissues regardless of obesity. Interestingly, the important regulatory enzyme inhibiting glycogen synthase activity and thus regulating glycogen metabolism, glycogen synthase kinase-3 (GSK-3), showed significantly higher protein expression levels in malignant tumor tissues of normal-weight women compared to either benign tumor tissue or malignant tumor tissue from overweight/obese-weight women (Figure 1G). This finding may be indicative of context-dependent utilization of glucose depositions by breast cancer tissue, most likely representing an adaptive mechanism that is lost in malignant tissue of overweight/obese-weight women. 

### 3.2. Breast Cancer Activates Energy Metabolism in Tumor-Associated Adipose Tissue and Induces Profound Reprogramming of Glucose Metabolism

**Figure 2:**
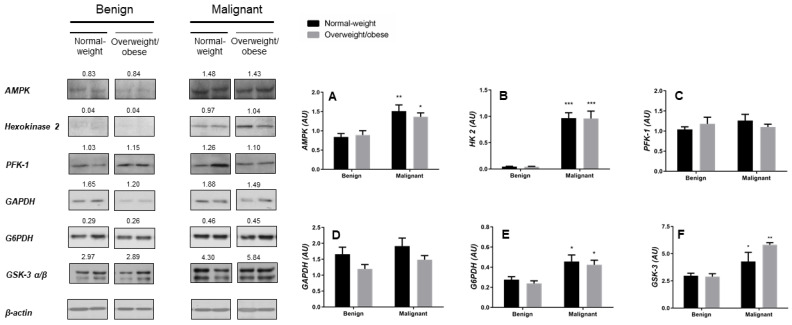
Breast cancer-associated adipose tissue changes in glucose metabolism irrespective of obesity. Protein expression of 5′-AMP-activated protein kinase (AMPK, **A**), hexokinase 2 (HK 2, **B**), phosphofructokinase (PFK-1, **C**), glyceraldehyde-3-phosphate dehydrogenase (GAPDH, **D**), glucose-6-phosphate 1-dehydrogenase (G6PDH, **E**), and glycogen synthase kinase-3 (GSK-3 α/β, **F**) in benign and malignant tumor-associated adipose tissue of normal-weight (black) and overweight/obese (gray) women. The protein content is normalized to β-actin levels and expressed in arbitrary units (AU); mean values of densitometric analysis for each group are shown above respective bands. Two representative band images cut from the same blot are shown for each group (*n* = 9); each band is representative of three pooled samples. Images of whole uncut blots with values of densitometric analysis for each band are shown Figure S1. Bars represent the average values ± S.E.M. * Comparison to respective benign counterpart, * *p* < 0.05, ** *p* < 0.01, *** *p* < 0.001.

To investigate the metabolic features of cancer-associated adipose tissue from normal-weight and overweight/obese women, protein expression analysis of key metabolic enzymes in glucose metabolism was performed using western blotting. In comparison to the breast adipose tissue of women with benign tumors, malignant tumor-associated adipose tissue showed higher protein expression levels of AMPK and adipocyte-specific isoform of hexokinase—HK 2 (Figure 2A,B), while protein expression of downstream glycolytic enzymes PFK-1 and GAPDH did not change (Figure 2C,D). These data indicate that metabolic reprogramming and increased glucose utilization in tumor-associated adipose tissue are not directed towards pyruvate production but rather towards the pentose phosphate pathway as supported by a higher protein expression of G6PDH observed (Figure 2E). Interestingly, protein expression of GSK-3 (Figure 2F) was also higher in cancer-associated adipose tissue compared to adipose tissue of women with benign breast tumors. No obesity-associated differences in protein expression of metabolic enzymes were found in adipose tissue of premenopausal women with breast cancer.

### 3.3. Glycogen Content Reveals Obesity-Related Differences in Glucose Metabolism between Malignant Tumor Tissues of Normal-Weight and Overweight/Obese Premenopausal Women

**Figure 3:**
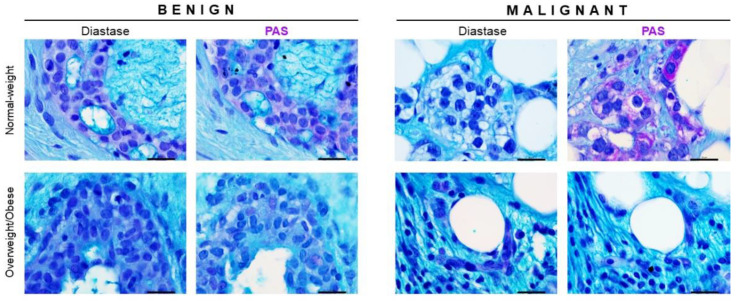
Glycogen content changes in breast cancer tissue are associated with obesity. Periodic Acid-Schiff (PAS) staining and light microscopy showing glycogen localization patterns in benign and malignant tumor tissues. Glycogen (purple staining) localization in breast cancer cells in malignant tumor tissue and breast epithelial cells in benign tumor tissue of normal-weight and overweight/obese premenopausal women. For each sample, PAS staining is presented next to the serial section pretreated with diastase, an enzyme that catalyzes glycogen hydrolysis. Magnification: ×100, orig. Scale bar: 20 μm.

To assess the glycogen content and analyze glycogen localization in tumor tissues, we performed periodic Acid-Schiff (PAS) staining. Glycogen deposition was observed in benign tumor tissues of both normal-weight and overweight/obese women and was primarily localized in breast epithelial cells (Figure 3). Interestingly, malignant tumor tissues of normal-weight women showed strong cytoplasmic staining for glycogen in breast cancer cells, while no prominent glycogen deposition was observed in breast cancer cells in malignant tumor tissue of overweight/obese women (Figure 3). These results may indicate important obesity-related differences in glycogen metabolism.

### 3.4. Cell-Specific Localization of Hexokinase 2 in Cancer Cells and Cancer-Associated Adipocytes in Breast Cancer of Normal-Weight and Overweight/Obese Premenopausal Women

**Figure 4:**
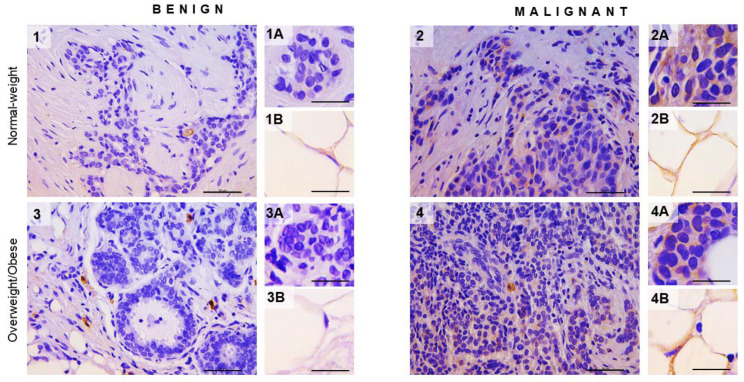
Hexokinase 2 expression in breast tumor and associated adipose tissue in normal-weight and overweight/obesepremenopausal women. Immunohistochemistry and light microscopy showing hexokinase 2 protein localization patterns in benign (panels 1 and 3) and malignant (panels 2 and 4) tumor tissue. Cell-specific hexokinase 2 (brown staining) protein localization in breast epithelial cells (inserts **1A** and **3A**) and adipocytes (**1B** and **3B**) in benign tumor tissues and breast cancer cells (inserts **2A** and **4A**) and cancer-associated adipocytes (**2B** and **4B**) in malignant tumor tissues of normal-weight (panels 1 and 2) and overweight/obese (panels 3 and 4) premenopausal women. Magnification: ×40 and ×100, orig., respectively. Scale bars: 50 μm and 20 μm, respectively.

We performed immunohistochemistry on tissue sections of benign and malignant breast tumor samples from normal-weight and overweight/obese individuals to assess any changes in HK 2 expression in tumor tissues and confirm adipocyte cell-specific localization of HK 2 in tumor-associated adipose tissue. As expected, breast cancer cells showed strong immunopositivity for HK 2 compared to benign tissues in both normal-weight and overweight/obese individuals (Figure 4). Moreover, adipocytes were also observed to express HK 2 in malignant tumor-associated adipose tissue irrespective of obesity, but a more prominent immunopositivity was observed at the invasive front of the tumor. Overexpression of HK 2 in tumor-associated adipose tissue was quite surprising and has not been reported before as a part of an adipose tissue metabolic reprogramming in malignancy.

### 3.5. Functional Localization of Hexokinase 2 in Mitochondria of Breast Cancer Tissue

**Figure 5:**
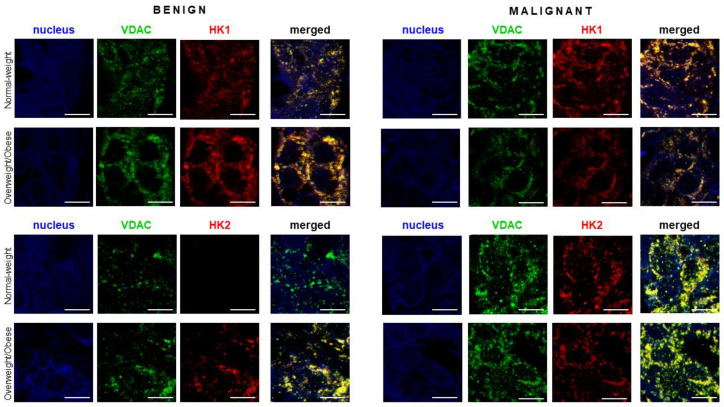
Hexokinase 2 colocalization with mitochondrial marker VDAC in premenopausal breast cancer. Immunofluorescence staining and confocal microscopy images showing voltage-dependent anion-selective channel protein 1 (VDAC)/hexokinase 1 (HK 1) and VDAC/hexokinase 2 (HK 2) protein localization in benign and malignant breast tumor tissue of normal-weight and overweight/obese premenopausal women. Double immunolabeling is shown in separate channel micrographs against VDAC (green), HKs (red), and as merged channels showing colocalization of VDAC and HKs (yellow). Nuclei were stained with Sytox Orange (false blue). Magnification: ×63, Zoom: ×2. Scale bars: 10 μm.

To analyze if metabolic reprogramming involving overexpression of HK 2 in malignant tissues is associated with its functional translocation to mitochondria, we analyzed colocalization patterns of HK 1 and HK 2 in relation to VDAC, a mitochondrial marker by immunofluorescent staining and confocal microscopy (Figure 5). The immunocytochemistry results support the protein expression data obtained by western blotting. As expected, HK 1 was constitutively expressed in all tumor tissues regardless of malignancy and obesity and colocalized with VDAC. For HK 2, immunofluorescence staining showed strong induction of HK 2 expression in malignant tumor tissues of normal-weight women compared to corresponding benign tumor tissues. Furthermore, HK 2 strongly colocalized with VDAC and was localized in mitochondria. Surprisingly, we also observed limited induction of HK 2 in benign tumor tissue of overweight/obese women.

## 4. Discussion

This study evaluated hallmark signatures of glucose metabolism in breast cancer and cancer-associated adipose tissue of normal-weight and overweight/obese premenopausal women. Regardless of obesity, increased AMPK protein expression was found in both breast cancer tissue and cancer-associated adipose tissue, synchronous with the induction of glucose utilization. Indeed, breast cancer tissue showed HK 2 overexpression and its association with a mitochondrial marker VDAC followed by increased PFK-1 and G6PDH protein expression. These changes indicate the induction of specific Warburg effect favoring glycolysis and pentose phosphate pathway, respectively. In parallel, cancer-associated adipose tissue showed overexpression of HK 2 and G6PDH, indicating metabolic reprogramming favoring the pentose phosphate pathway. Interestingly, some aspects of glucose metabolism differed depending on obesity (i.e., between normal-weight and overweight/obese premenopausal women). Breast cancer tissue showed abundant cytoplasmic glycogen deposition and higher GSK-3 protein expression in normal-weight women. In comparison, breast cancer tissue showed no prominent glycogen deposition but displayed higher PFK-1 and GAPDH protein expression in overweight/obese women. These results suggest context-dependent metabolic reprogramming in breast cancer tissue and the induction of tissue-specific Warburg effect in cancer and associated adipose tissue.

### 4.1. A Metabolic Sensor AMPK Is Overexpressed Simultaneously in Breast Cancer Tissue and Cancer-Associated Adipose Tissue

AMPK is a metabolic sensor and a master regulator of energy homeostasis whose activation stimulates catabolism acting on glucose uptake, glycolysis, oxygen consumption, fatty acid oxidation, lipolysis, and mitochondrial biogenesis [31,32,33,34]. The role of AMPK in cancer progression is rather complex. Previous studies on different cancer types have emphasized the anti-tumorigenic role of AMPK [35], partly through negative regulation of glycolysis and the Warburg effect [36]. However, recent data indicate that physiological activation of AMPK can contribute to tumor growth through maintenance of energy and redox homeostasis [37,38,39]. Moreover, AMPK activation may result in a specific Warburg effect that allows for metabolic plasticity in terms of simultaneous upregulation of glycolysis and the pentose phosphate pathway [40]. Namely, AMPK may support the Warburg effects by stimulating glucose uptake and glycolytic flux acting indirectly on GLUT1 [41] and PFK-1 [42] while maintaining pentose phosphate pathway flux and redox homeostasis through stimulation of G6PDH expression via histone deacetylase 10 [43]. In addition, the clinical significance of AMPK protein expression levels in breast cancer remains controversial. Several previous studies showed increased AMPK protein expression and a positive correlation with breast cancer type and progression stage [44,45], while others have found decreased AMPK expression and inverse associations [46]. Here, we showed increased AMPK protein expression in breast cancer tissue of premenopausal women, regardless of obesity. Interestingly, we observed a parallel increase in AMPK protein expression in cancer-associated adipose tissue providing additional evidence for cancer-induced metabolic reprogramming in adipose tissue. This is a novel finding on human tissue samples, with one previous in vitro study showing that breast cancer cells induce metabolically activated phenotype in adipocytes by activating AMPK signaling [47]. Parallel activation of AMPK in cancer and adipose tissue may serve to coordinate metabolic cooperation such as lipid trafficking. Separate studies showed that AMPK activation induces fatty acid oxidation in adipocytes, while adipocytes may secrete exosomes containing fatty acids and fatty acid oxidation proteins to promote cancer aggressiveness [48]. Additionally, adipocytes and adipose-derived fatty acids were shown to activate AMPK and fatty acid oxidation in cancer cells [49]. Further studies are clearly warranted to detangle these complex relationships between cancer cells and cancer-associated adipose tissue.

### 4.2. Tissue-Specific Warburg Effect Involving Overexpression of HK 2 Is Induced in Breast Cancer Tissue and Cancer-Associated Adipose Tissue

The Warburg effect refers to specific metabolic reprogramming where cancer cells rely on increased glucose uptake and glycolytic flux with lactate production even in the presence of oxygen to support growth and proliferation [50]. Overexpression and mitochondrial localization of HK 2 are the main features that enable such a long-lasting glycolytic phenotype [12]. Our study reveals that breast cancer tissue of premenopausal women shows markedly higher HK 2 protein expression and strong colocalization with a mitochondrial protein VDAC compared to benign breast tumor tissues irrespective of obesity. This is in line with previous research showing HK 2 protein expression and activity have been linked to histological grade, invasiveness, aggressiveness, adverse disease outcome, and shorter disease-free survival in breast cancer patients [51,52]. Hence, HK 2 was proposed as an attractive target for anti-cancer therapy [53,54,55]. Surprisingly, we observed some HK 2 immunoreactivity in benign tumor tissue of overweight/obese women that was absent in normal-weight women. Furthermore, increased PFK-1 and G6PDH protein expression in malignant tissues of normal-weight and overweight/obese women support a firm breast cancer tissue reliance on glucose catabolism for energy production, intermediate metabolism, and redox homeostasis. In addition, we found increased expression of HK 2 in cancer-associated adipose tissue indicating that malignancy promotes the Warburg effects in both cancer cells and cancer-associated adipocytes. Overexpression of HK 2 in cancer-associated adipose tissue was associated with an increase in G6PDH protein expression, indicating that glucose is primarily shuttled towards the pentose phosphate pathway. Thus, increased AMPK signaling and glucose utilization may support other metabolic pathways such as lipid metabolism and redox homeostasis maintenance [32]. Interestingly, HK 2 protein expression in benign breast tumor tissue of overweight/obese individuals and in cancer-associated adipose tissue may prove to be an important predictive marker. Indeed, chronic social isolation stress has been found to increase HK 2 gene expression, specifically in mammary adipose tissue, making the breast microenvironment more amenable for cancer progression [56]. Moreover, we found higher GSK-3 protein expression in cancer-associated adipose tissue. This is the first time changes in GSK-3 protein expression are being reported as part of the metabolic reprogramming of cancer-associated adipose tissue. However, previous studies showed that GSK-3 plays an important part in adipogenesis and adipose tissue metabolic reprogramming associated with obesity and insulin resistance [57,58,59].

### 4.3. Obesity Reprograms Specific Features of Glucose Metabolism in Breast Cancer Tissue

Our results show that some aspects of glucose metabolism in breast cancer tissues are specific to the obesity of premenopausal women. Namely, cross-examination of breast cancer tissues from normal-weight and overweight/obese premenopausal women revealed differences in protein markers of glycolysis and glycogen metabolism. Breast cancer tissues of normal-weight women showed abundant cytoplasmic glycogen deposition and increased GSK-3 protein expression compared to overweight/obese women, possibly indicating important obesity-related differences in glycogen metabolism. Previous studies showed cytoplasmic glycogen accumulation in breast cancer [60]. Additionally, PAS staining was shown to be potentially useful in differentiating benign and malignant breast changes [61], while PAS staining pattern in lymph node metastasis was associated with distant metastasis occurrence [62]. Dysregulated glycogen metabolism has been shown to contribute to various aspects of neoplastic transformation, from redox regulation and cancer-stroma interactions to proliferation, metastasis, and chemoresistance [63,64,65]. A recent in vitro study on ovarian cancer indicated a possible relationship between glycogen metabolism and cancer proliferation and metastasis, partly through stimulation of glycolysis and lactate production [66]. We recently showed that breast cancer tissues in normal-weight women have significantly higher tissue lactate concentration than those in obese women, unrelated to LDH activity and protein expression [25], which could be related to the more pronounced glycogen deposition as shown in this study. In contrast, breast cancer tissues of overweight/obese women showed higher PFK-1 and GAPDH protein expression compared to breast cancer tissues of normal-weight women. Accumulating evidence links worse breast cancer prognosis with higher expression and activity of glycolytic enzymes. PFK-1 activity increases with the tumor size and positively correlates with markers of breast cancer invasiveness and aggressiveness [51], while high gene expression of platelet type phosphofructokinase 1 correlates with decreased patient survival [67]. Similarly, GAPDH gene expression correlates with breast cancer grade, overall survival, and disease-free survival [68]. Obesity-dependent differences in cancer tissue glucose metabolism presented in our study likely reflect context-dependent features of metabolic reprogramming and may prove to be valuable targets for diagnostics and the design of personalized therapeutic approaches. Interestingly, these differences may reflect systemic, rather than local, influences of obesity since we did not detect obesity-related differences in glucose metabolism in cancer-associated adipose tissue. Lack of differences between normal-weight and overweight/obese women with either benign or malignant breast tumors may be related to depot-specificity of mammary adipose tissue and specificity of our study cohort. Mammary adipose tissue is a special type of subcutaneous adipose tissue, known for its morpho-functional plasticity, especially during pregnancy and lactation [69,70]. In general, changes in adipose tissue glucose metabolism were previously associated with obesity, insulin resistance, and diabetes, but these changes were more prominent in visceral adipose tissue depots [71,72]. Moreover, our study cohort was comprised of young, premenopausal, insulin-sensitive, and mainly overweight women. Mammary adipose tissue functions previously associated with breast cancer progression and obesity, such as lipolysis, secretion of growth factors, adipokines, and proinflammatory cytokines, were mainly studied in vitro [73,74] or were associated with age, menopausal status, and insulin resistance in vivo [75]. More in vivo and human studies are necessary to reveal obesity-related changes to the fundamental aspects of mammary adipose tissue metabolism, especially in premenopausal women.

## 5. Conclusions

This study evaluated protein expression of AMPK, key rate-limiting and regulatory enzymes of glycolysis, pentose phosphate pathway, and glycogen metabolism in breast cancer and cancer-associated adipose tissue in normal-weight and overweight/obese premenopausal women. We show the simultaneous induction of AMPK protein expression and tissue-specific Warburg effects in breast cancer and associated adipose tissue, opening the question of novel roles for AMPK in glycolytic reprogramming in cancer. Namely, increased glucose utilization favors glycolysis and pentose phosphate pathway in breast cancer tissue, with typical features of the Warburg effects including overexpression and mitochondrial localization of HK 2. In contrast, increased glucose utilization in adipose tissue mainly favors the pentose phosphate pathway. Moreover, certain aspects of glucose metabolism seem to be obesity-dependent. Breast cancer tissue in normal-weight women shows more pronounced glycogen deposition, while breast cancer tissue in overweight/obese shows higher protein expression of PFK-1 and GAPDH glycolytic enzymes. Parallel reprogramming of cancer cells and cancer-associated adipose tissue down specific metabolic phenotypes may reveal new aspects of metabolic coupling in breast cancer. Moreover, important obesity-related changes to the glucose metabolism of breast cancer tissue could inform the discovery of novel targets for personalized diagnostic and therapeutic approaches. Our future studies will be directed towards revealing the mechanism of possible AMPK involvement in glycolytic reprogramming as well as fatty acid metabolism and oxidative phosphorylation in breast cancer and cancer-associated adipose tissue.

## Data Availability

The data presented in this study are available on reasonable request from the corresponding author.

## Supplementary Materials

The following are available online at https://www.mdpi.com/article/10.3390/cancers13112731/s1, Figure S1: Western blot.

## References

[1] Heer E., Harper A., Escandor N., Sung H., McCormack V., Fidler-Benaoudia M.M. (2020). Global burden and trends in premenopausal and postmenopausal breast cancer: A population-based study. Lancet Glob. Health.

[2] Nichols H.B., Schoemaker M.J., Wright L.B., McGowan C., Brook M.N., Mcclain K.M., Jones M.E., Adami H.O., Agnoli C., Baglietto L. (2017). The premenopausal breast cancer collaboration: A pooling project of studies participating in the national cancer institute cohort consortium. Cancer Epidemiol. Biomark. Prev..

[3] Rosenberg S.M., Partridge A.H. (2015). Management of breast cancer in very young women. Breast.

[4] Lewis D.R., Seibel N.L., Smith A.W., Stedman M.R. (2014). Adolescent and young adult cancer survival. J. Natl. Cancer Inst. Monogr..

[5] Engin A. (2017). Obesity-associated breast cancer: Analysis of risk factors. Adv. Exp. Med. Biol..

[6] Schoemaker M.J., Nichols H.B., Wright L.B., Brook M.N., Jones M.E., O’Brien K.M., Adami H.O., Baglietto L., Bernstein L., Bertrand K.A. (2018). Association of body mass index and age with subsequent breast cancer risk in premenopausal women. JAMA Oncol..

[7] Chan D.S.M., Vieira A.R., Aune D., Bandera E.V., Greenwood D.C., McTiernan A., Navarro Rosenblatt D., Thune I., Vieira R., Norat T. (2014). Body mass index and survival in women with breast cancer—Systematic literature review and meta-analysis of 82 follow-up studies. Ann. Oncol..

[8] Niraula S., Ocana A., Ennis M., Goodwin P.J. (2012). Body size and breast cancer prognosis in relation to hormone receptor and menopausal status: A meta-analysis. Breast Cancer Res. Treat..

[9] Lee K., Kruper L., Dieli-Conwright C.M., Mortimer J.E. (2019). The impact of obesity on breast cancer diagnosis and treatment. Curr. Oncol. Rep..

[10] Hay N. (2016). Reprogramming glucose metabolism in cancer: Can it be exploited for cancer therapy?. Nat. Rev. Cancer.

[11] Warburg O. (1956). On the origin of cancer cells. Science.

[12] Pedersen P.L. (2007). Warburg, me and Hexokinase 2: Multiple discoveries of key molecular events underlying one of cancers’ most common phenotypes, the “Warburg Effect”, i.e., elevated glycolysis in the presence of oxygen. J. Bioenerg. Biomembr..

[13] Mathupala S.P., Ko Y.H., Pedersen P.L. (2009). Hexokinase-2 bound to mitochondria: Cancer’s stygian link to the “Warburg effect” and a pivotal target for effective therapy. Semin. Cancer Biol..

[14] John S., Weiss J.N., Ribalet B. (2011). Subcellular localization of hexokinases I and II directs the metabolic fate of glucose. PLoS ONE.

[15] Nakashima R.A., Pedersen P.L., Mangan P.S., Colombini M. (1986). Hexokinase receptor complex in hepatoma mitochondria: Evidence from N,N′-Dicyclohexylcarbodiimide-labeling studies for the involvement of the pore-forming protein VDAC. Biochemistry.

[16] Bose S., Le A. (2018). Glucose metabolism in cancer. Adv. Exp. Med. Biol..

[17] Jin L., Zhou Y. (2019). Crucial role of the pentose phosphate pathway in malignant tumors (review). Oncol. Lett..

[18] Dias A.S., Almeida C.R., Helguero L.A., Duarte I.F. (2019). Metabolic crosstalk in the breast cancer microenvironment. Eur. J. Cancer.

[19] Soysal S.D., Tzankov A., Muenst S.E. (2015). Role of the tumor microenvironment in breast cancer. Pathobiology.

[20] Zhao C., Wu M., Zeng N., Xiong M., Hu W., Lv W., Yi Y., Zhang Q., Wu Y. (2020). Cancer-associated adipocytes: Emerging supporters in breast cancer. J. Exp. Clin. Cancer Res..

[21] Lengyel E., Makowski L., DiGiovanni J., Kolonin M.G. (2018). Cancer as a Matter of Fat: The Crosstalk between Adipose Tissue and Tumors. Trends Cancer.

[22] Gupta S., Roy A., Dwarakanath B.S. (2017). Metabolic cooperation and competition in the tumor microenvironment: Implications for therapy. Front. Oncol..

[23] Li F., Simon M.C. (2020). Cancer cells don’t live alone: Metabolic communication within tumor microenvironments. Dev. Cell.

[24] Wu Q., Li B., Li Z., Li J., Sun S., Sun S. (2019). Cancer-associated adipocytes: Key players in breast cancer progression. J. Hematol. Oncol..

[25] Kalezic A., Udicki M., Galic B.S., Aleksic M., Korac A., Jankovic A., Korac B. (2020). Lactate metabolism in breast cancer microenvironment: Contribution focused on associated adipose tissue and obesity. Int. J. Mol. Sci..

[26] Kalezic A., Udicki M., Galic B.S., Aleksic M., Korac A., Jankovic A., Korac B. (2021). Redox profile of breast tumor and associated adipose tissue in premenopausal women—Interplay between obesity and malignancy. Redox Biol..

[27] WHO (2000). Obesity: Preventing and Managing the Global Epidemic: Report of a WHO Consultation.

[28] Petrović V., Korać A., Buzadžić B., Korać B. (2005). The effects of L-arginine and L-NAME supplementation on redox-regulation and thermogenesis in interscapular brown adipose tissue. J. Exp. Biol..

[29] Jankovic A., Golic I., Markelic M., Stancic A., Otasevic V., Buzadzic B., Korac A., Korac B. (2015). Two key temporally distinguishable molecular and cellular components of white adipose tissue browning during cold acclimation. J. Physiol..

[30] Golic I., Kalezic A., Jankovic A., Jonic S., Korac B., Korac A. (2020). Insulin modulates the bioenergetic and thermogenic capacity of rat brown adipocytes in vivo by modulating mitochondrial mosaicism. Int. J. Mol. Sci..

[31] Wang L., Di L., Noguchi C.T. (2014). AMPK is involved in mediation of erythropoietin influence on metabolic activity and reactive oxygen species production in white adipocytes. Int. J. Biochem. Cell Biol..

[32] Bijland S., Mancini S.J., Salt I.P. (2013). Role of AMP-activated protein kinase in adipose tissue metabolism and inflammation. Clin. Sci..

[33] Kim S.-J., Tang T., Abbott M., Viscarra J.A., Wang Y., Sul H.S. (2016). AMPK phosphorylates Desnutrin/ATGL and hormone-sensitive lipase to regulate lipolysis and fatty acid oxidation within adipose tissue. Mol. Cell. Biol..

[34] Grabacka M., Pierzchalska M., Dean M., Reiss K. (2016). Regulation of ketone body metabolism and the role of PPARα. Int. J. Mol. Sci..

[35] Zadra G., Batista J.L., Loda M. (2015). Dissecting the dual role of AMPK in cancer: From experimental to human studies. Mol. Cancer Res..

[36] Faubert B., Boily G., Izreig S., Griss T., Samborska B., Dong Z., Dupuy F., Chambers C., Fuerth B.J., Viollet B. (2013). AMPK is a negative regulator of the warburg effect and suppresses tumor growth in vivo. Cell Metab..

[37] Jeon S.-M., Hay N. (2012). The dark face of AMPK as an essential tumor promoter. Cell. Logist..

[38] Doménech E., Maestre C., Esteban-Martínez L., Partida D., Pascual R., Fernández-Miranda G., Seco E., Campos-Olivas R., Pérez M., Megias D. (2015). AMPK and PFKFB3 mediate glycolysis and survival in response to mitophagy during mitotic arrest. Nat. Cell Biol..

[39] Jeon S.M., Chandel N.S., Hay N. (2012). AMPK regulates NADPH homeostasis to promote tumour cell survival during energy stress. Nature.

[40] Laderoute K.R., Calaoagan J.M., Chao W.R., Dinh D., Denko N., Duellman S., Kalra J., Liu X., Papandreou I., Sambucetti L. (2014). 5′-AMP-activated protein kinase (AMPK) supports the growth of aggressive experimental human breast cancer tumors. J. Biol. Chem..

[41] Wu N., Zheng B., Shaywitz A., Dagon Y., Tower C., Bellinger G., Shen C.H., Wen J., Asara J., McGraw T.E. (2013). AMPK-dependent degradation of TXNIP upon energy stress leads to enhanced glucose uptake via GLUT1. Mol. Cell.

[42] Marsin A.S., Bertrand L., Rider M.H., Deprez J., Beauloye C., Vincent M.F., Van den Berghe G., Carling D., Hue L. (2000). Phosphorylation and activation of heart PFK-2 by AMPK has a role in the stimulation of glycolysis during ischaemia. Curr. Biol..

[43] Shan C., Lu Z., Li Z., Sheng H., Fan J., Qi Q., Liu S., Zhang S. (2019). 4-hydroxyphenylpyruvate dioxygenase promotes lung cancer growth via pentose phosphate pathway (PPP) flux mediated by LKB1-AMPK/HDAC10/G6PD axis. Cell Death Dis..

[44] Al-Maghrabi J., Al-Sakkaf K., Qureshi I.A., Butt N.S., Damnhory L., Elshal M., Al-Maghrabi B., Aldahlawi A., Ashoor S., Brown B. (2017). AMPK expression patterns are significantly associated with poor prognosis in breast cancer patients. Ann. Diagn. Pathol..

[45] Liu P., Ye F., Xie X., Li X., Tang H., Li S., Huang X., Song C., Wei W., Xie X. (2016). mir-101-3p is a key regulator of tumor metabolism in triple negative breast cancer targeting AMPK. Oncotarget.

[46] Fox M.M., Phoenix K.N., Kopsiaftis S.G., Claffey K.P. (2013). AMP-activated protein kinase α 2 isoform suppression in primary breast cancer alters AMPK growth control and apoptotic signaling. Genes Cancer.

[47] Sun S., Wu Q., Li J., Li Z., Sun S., Zhu S., Wang L., Wu J., Yuan J., Zhang Y. (2019). Exosomes from the tumour-adipocyte interplay stimulate beige/brown differentiation and reprogram metabolism in stromal adipocytes to promote tumour progression. J. Exp. Clin. Cancer Res..

[48] Lazar I., Clement E., Dauvillier S., Milhas D., Ducoux-Petit M., LeGonidec S., Moro C., Soldan V., Dalle S., Balor S. (2016). Adipocyte exosomes promote melanoma aggressiveness through fatty acid oxidation: A novel mechanism linking obesity and cancer. Cancer Res..

[49] Wen Y.A., Xing X., Harris J.W., Zaytseva Y.Y., Mitov M.I., Napier D.L., Weiss H.L., Mark Evers B., Gao T. (2017). Adipocytes activate mitochondrial fatty acid oxidation and autophagy to promote tumor growth in colon cancer. Cell Death Dis..

[50] Heiden M.G.V., Cantley L.C., Thompson C.B. (2009). Understanding the warburg effect: The metabolic requirements of cell proliferation. Science.

[51] Coelho R.G., Calaça I.C., Celestrini D.M., Correia-Carneiro A.H.P., Costa M.M., Zancan P., Sola-Penna M. (2015). Hexokinase and phosphofructokinase activity and intracellular distribution correlate with aggressiveness and invasiveness of human breast carcinoma. Oncotarget.

[52] Sato-Tadano A., Suzuki T., Amari M., Takagi K., Miki Y., Tamaki K., Watanabe M., Ishida T., Sasano H., Ohuchi N. (2013). Hexokinase II in breast carcinoma: A potent prognostic factor associated with hypoxia-inducible factor-1α and Ki-67. Cancer Sci..

[53] Marini C., Salani B., Massollo M., Amaro A., Esposito A.I., Orengo A.M., Capitanio S., Emionite L., Riondato M., Bottoni G. (2013). Direct inhibition of hexokinase activity by metformin at least partially impairs glucose metabolism and tumor growth in experimental breast cancer. Cell Cycle.

[54] Lis P., Dylag M., Niedźwiecka K., Ko Y.H., Pedersen P.L., Goffeau A., Ułaszewski S. (2016). The HK2 dependent “Warburg effect” and mitochondrial oxidative phosphorylation in cancer: Targets for effective therapy with 3-bromopyruvate. Molecules.

[55] Chen Z., Zhang H., Lu W., Huang P. (2009). Role of mitochondria-associated hexokinase II in cancer cell death induced by 3-bromopyruvate. Biochim. Biophys. Acta Bioenerg..

[56] Volden P.A., Wonder E.L., Skor M.N., Carmean C.M., Patel F.N., Ye H., Kocherginsky M., McClintock M.K., Brady M.J., Conzen S.D. (2013). Chronic social isolation is associated with metabolic gene expression changes specific to mammary adipose tissue. Cancer Prev. Res..

[57] Eldar-Finkelman H., Schreyer S.A., Shinohara M.M., LeBoeuf R.C., Krebs E.G. (1999). Increased glycogen synthase kinase-3 activity in diabetes- and obesity- prone C57BL/6J mice. Diabetes.

[58] Chakraborty A., Koldobskiy M.A., Bello N.T., Maxwell M., Potter J.J., Juluri K.R., Maag D., Kim S., Huang A.S., Dailey M.J. (2010). Inositol pyrophosphates inhibit akt signaling, thereby regulating insulin sensitivity and weight gain. Cell.

[59] Yan C., Yang H., Wang Y., Dong Y., Yu F., Wu Y., Wang W., Adaku U., Lutfy K., Friedman T.C. (2016). Increased glycogen synthase kinase-3β and hexose-6-phosphate dehydrogenase expression in adipose tissue may contribute to glucocorticoid-induced mouse visceral adiposity. Int. J. Obes..

[60] Altemus M.A., Goo L.E., Little A.C., Yates J.A., Cheriyan H.G., Wu Z.F., Merajver S.D. (2019). Breast cancers utilize hypoxic glycogen stores via PYGB, the brain isoform of glycogen phosphorylase, to promote metastatic phenotypes. PLoS ONE.

[61] Panicker N.K., Jariwala P.H., Buch A.C., Joshi M. (2012). The utility of periodic acid schiff with diastase and alcian blue stains on fine needle aspirates of breast and salivary gland neoplasms. J. Cytol..

[62] Buijs J.T., Cleton A.M., Smit V.T.H.B.M., Löwik C.W.G.M., Papapoulos S.E., Van Der Pluijm G. (2004). Prognostic significance of periodic acid-Schiff-positive patterns in primary breast cancer and its lymph node metastases. Breast Cancer Res. Treat..

[63] Khan T., Sullivan M.A., Gunter J.H., Kryza T., Lyons N., He Y., Hooper J.D. (2020). Revisiting glycogen in cancer: A conspicuous and targetable enabler of malignant transformation. Front. Oncol..

[64] Shulman R.G., Rothman D.L. (2017). The glycogen shunt maintains glycolytic homeostasis and the Warburg effect in cancer. Trends Cancer.

[65] Zois C.E., Harris A.L. (2016). Glycogen metabolism has a key role in the cancer microenvironment and provides new targets for cancer therapy. J. Mol. Med..

[66] Curtis M., Kenny H.A., Ashcroft B., Mukherjee A., Johnson A., Zhang Y., Helou Y., Batlle R., Liu X., Gutierrez N. (2019). Fibroblasts mobilize tumor cell glycogen to promote proliferation and metastasis. Cell Metab..

[67] Prasad C.P., Södergren K., Andersson T. (2017). Reduced production and uptake of lactate are essential for the ability of WNT5A signaling to inhibit breast cancer cell migration and invasion. Oncotarget.

[68] Révillion F., Pawlowski V., Hornez L., Peyrat J.P. (2000). Glyceraldehyde-3-phosphate dehydrogenase gene expression in human breast cancer. Eur. J. Cancer.

[69] Colleluori G., Perugini J., Barbatelli G., Cinti S. (2021). Mammary gland adipocytes in lactation cycle, obesity and breast cancer. Rev. Endocr. Metab. Disord..

[70] Wronska A., Kmiec Z. (2012). Structural and biochemical characteristics of various white adipose tissue depots. Acta Physiol..

[71] Jankovic A., Korac A., Buzadzic B., Otasevic V., Stancic A., Daiber A., Korac B. (2015). Redox implications in adipose tissue (dys)function—A new look at old acquaintances. Redox Biol..

[72] Sethi J.K., Vidal-Puig A.J. (2007). Thematic review series: Adipocyte biology. Adipose tissue function and plasticity orchestrate nutritional adaptation. J. Lipid Res..

[73] Balaban S., Shearer R.F., Lee L.S., van Geldermalsen M., Schreuder M., Shtein H.C., Cairns R., Thomas K.C., Fazakerley D.J., Grewal T. (2017). Adipocyte lipolysis links obesity to breast cancer growth: Adipocyte-derived fatty acids drive breast cancer cell proliferation and migration. Cancer Metab..

[74] D’Esposito V., Passaretti F., Hammarstedt A., Liguoro D., Terracciano D., Molea G., Canta L., Miele C., Smith U., Beguinot F. (2012). Adipocyte-released insulin-like growth factor-1 is regulated by glucose and fatty acids and controls breast cancer cell growth in vitro. Diabetologia.

[75] Kolb R., Zhang W. (2020). Obesity and breast cancer: A case of inflamed adipose tissue. Cancers.

