# Supplementary material for: Tissue-Specific Warburg Effect in Breast Cancer and Cancer-Associated Adipose Tissue—Relationship between AMPK and Glycolysis
Source: Cancers (Basel). 2021 May 31;13(11):2731. doi: 10.3390/cancers13112731 (PMC8198826; doi:10.3390/cancers13112731)

Western blot

Supplementary Figure S1. Images of whole blots against 5'-AMP-activated protein kinase catalytic subunit alpha-1 (AMPK), hexokinase I (HK I), hexokinase II (HK II), phosphofructokinase 1 (PFK-1), glyceraldehyde-3-phosphate dehydrogenase (GAPDH), glucose-6-phosphate 1-dehydrogenase (G6PDH), glycogen synthase kinase-3 (GSK-3 α/β), and β-actin for tumor and adipose tissue, respectively. Densitometric analysis of protein content normalized against β-actin is given in arbitrary units and is shown below each respective band. Each image is representative of three independent trials, showing three representative bands for tumor tissue and adipose tissue per group (normal-weight women with benign tumors, overweight/obese women with benign tumors, normal-weight women with malignant tumors, and overweight/obese women with malignant tumors, respectively). Prior to loading, nine samples from each group were pooled by three to obtain three samples shown in blots.

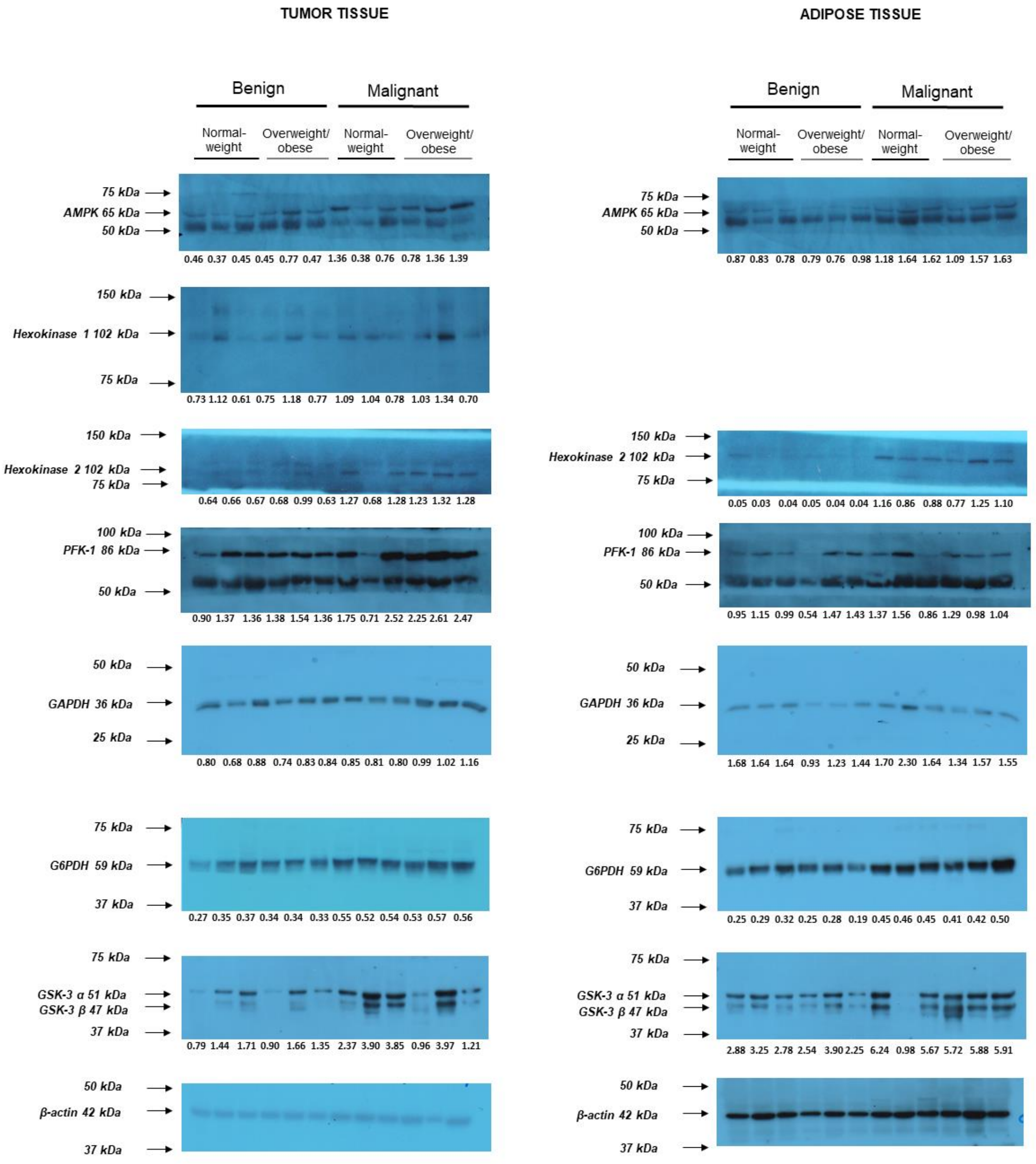

Supplement: Supplementary file 1 [file cancers-13-02731-s001.zip › cancers-1216393-supplementary.pdf]
